# Social Health Insurance-Based Simultaneous Screening for 154 Mutations in 19 Deafness Genes Efficiently Identified Causative Mutations in Japanese Hearing Loss Patients

**DOI:** 10.1371/journal.pone.0162230

**Published:** 2016-09-14

**Authors:** Kentaro Mori, Hideaki Moteki, Maiko Miyagawa, Shin-ya Nishio, Shin-ichi Usami

**Affiliations:** 1 Department of Otolaryngology, Shinshu University School of Medicine, Matsumoto, Japan; 2 Department of Hearing Implant Sciences, Shinshu University School of Medicine, Matsumoto, Japan; NIDCR/NIH, UNITED STATES

## Abstract

Sensorineural hearing loss is one of the most common neurosensory disorders in humans. The incidence of SNHL is estimated to be 1 in 500–1000 newborns. In more than half of these patients, the hearing loss is associated with genetic causes. In Japan, genetic testing for the patients with SNHL using the Invader assay to screen for 46 mutations in 13 deafness genes was approved by the Ministry of Health, Labour and Welfare for inclusion in social health insurance coverage in 2012. Furthermore, from August 2015, this genetic testing has been expanded to screen for 154 mutations in 19 deafness genes using targeted genomic enrichment with massively parallel DNA sequencing combined with the Invader assay and TaqMan genotyping. For this study we analyzed 717 unrelated Japanese hearing loss patients. The total allele frequency of 154 mutations in 19 deafness genes was 32.64% (468/1434) and the total numbers of cases associated with at least one mutation was 44.07% (316/717). Among these, we were able to diagnose 212 (30%) patients, indicating that the present screening could efficiently identify causative mutations in hearing loss patients. It is noteworthy that 27 patients (3.8%) had coexistent multiple mutations in different genes. Five of these 27 patients (0.7%, 5/717 overall) were diagnosed with genetic hearing loss affected by concomitant with responsible mutations in more than two different genes. For patients identified with multiple mutations in different genes, it is necessary to consider that several genes might have an impact on their phenotypes.

## Introduction

Sensorineural hearing loss (SNHL) is one of the most common neurosensory disorders in humans. The incidence of SNHL is estimated to be 1 in 500–1000 newborns [1,2]. The hearing loss in more than half of these patients is associated with genetic causes [1,2]. This form of hearing loss is extremely heterogeneous, with over 80 genes known to be responsible for non-syndromic hearing loss (NSHL—hearing loss in the absence of other phenotypic findings), although more than 140 loci have been mapped [3]. The genetic diagnosis of SNHL is becoming more important to its precise diagnosis for the prediction of the severity and progressiveness of the hearing loss as well as late-onset associated symptoms. Furthermore, such information is useful for the selection of an appropriate intervention.

In Japan, genetic testing for patients with SNHL using the Invader assay to screen for 46 mutations in 13 deafness genes was approved by the Ministry of Health, Labour and Welfare for inclusion in social health insurance coverage in 2012. Furthermore, from August 2015, this genetic testing has been expanded to screen for 154 mutations in 19 deafness genes using targeted genomic enrichment with massively parallel DNA sequencing (TGE+MPS) combined with the Invader assay and TaqMan genotyping. We have recently reported the efficacy of this genetic testing protocol; i.e., TGE+MPS combined with the Invader assay and TaqMan genotyping, in identifying the responsible genes, and also suggested that it offered economic benefits [4].

However, TGE+MPS analysis provides results for hundreds or even thousands of variants, including those of uncertain pathogenicity and variant annotation and interpretation of MPS data requires due caution.

From the point of view of social health insurance-based genetic testing, the cost-effectiveness and robustness of the results need to be considered. Therefore, we focused on the variants of 154 mutations in 19 deafness genes that have been identified and reported in the Japanese hearing loss population.

In this study, we aimed to show (1) the frequency of mutations by screening of 154 mutations in 19 deafness genes in Japanese hearing loss patients, (2) the efficacy of this protocol with regard to social health insurance-based genetic testing, and (3) the frequency of multiple mutations.

## Materials and Methods

### Subjects

We analyzed 717 DNA samples from unrelated Japanese hearing loss patients enrolled from 53 clinical centers nationwide. All subjects had presumed non-syndromic SNHL. In this study, written informed consent was obtained from all participants (proband and their affected and unaffected relatives). This study was approved by the ethical committee of Shinshu University and each participating institution as described previously [5]. Clinical information and blood samples were obtained from each proband and their affected and unaffected relatives.

### Methods

We used the Invader assay, TaqMan genotyping assay and TGE+MPS. There were two reasons why we performed the three tests simultaneously; first the Invader assay is superior in terms of cost for the detection of mutations in mitochondrial DNA with various heteroplasmy rates and, second, the TaqMan genotyping assay is able to detect *KCNQ4* c.211delC mutations that are technically difficult to identify using the TGE+MPS employed due to their location in the extremely GC-rich region.

#### Invader assay

We first applied the Invader assay to screen for 46 known mutations in 13 deafness genes based on the mutation spectrum in the Japanese deafness population, the detail procedure for which was described previously [6].

#### TaqMan genotyping assay

For additional screening, TaqMan genotyping assays for *KCNQ4* c.211delC were applied for all subjects. The detail procedure was described previously [5].

#### Amplicon Library Preparation

An Amplicon library of the target exons was prepared with an Ion AmpliSeq^™^ Custom Panel (Applied Biosystems, Life Technologies) for 63 genes reported to cause non-syndromic hearing loss [3]. The detailed protocol was described elsewhere [7].

#### Emulsion PCR and Sequencing

The emulsion PCR and MPS was performed with an Ion Torrent Personal Genome Machine (PGM) system using the Ion PGM^™^ 200 Sequencing Kit and Ion 318^™^ Chip (Life Technologies) according to the manufacturer’s instructions.

#### Base Call and Data Analysis

The sequence data were processed with standard Ion Torrent Suite^™^ Software ver 4.0 with the Hot Spot BED option as described previously [5]. After variant detection, variant effects were analyzed using the wANNOVAR web site [8,9].

## Results

### Frequency of the 154 mutations in 19 deafness genes

Variants identified using the three genetic tests are listed in S1 Table. The allele frequencies of each variant identified among the 717 patients and 269 controls are shown in Table 1. In the group of 717 hearing loss patients, the total allele frequency of 154 mutations in 19 deafness genes was 32.64%(468/1434). Mutations were most frequently found in the *GJB2 gene* (18.76%, 269/1434 alleles). The second most frequent mutation was in the *SLC26A4* gene (5.30%, 76/1434 alleles), followed by the *CDH23* gene (3.97%, 57/1434 alleles); this result was generally consistent with the results in previous reports [6,10,11]. In this study, no *EYA1*, *MYO7A*, or *POU3F4* gene mutations were detected in any of the 717 hearing loss patients. In the group of 269 controls, the total allele frequency was 5.39% (29/538 alleles), and the most common mutations in the controls were those in GJB2 (1.86%, 10/538 alleles). None of the controls in this study had hearing complications. The mutations identified from them indicated carrier status.

**Table 1:** Mutations found in the present study.

				Hearing loss patients (n = 717)		Control (n = 269)	
*Gene*	*NM number*	*Amino acid change*	*Nucleotide change*	*Frequency of mutant alleles (n = 1434)*	*Number of patients*	*Frequency of mutant alleles (n = 538)*	*Number of controls*
*GJB2*	NM_004004	p.M195V	c.583A>G	4 (0.28%)	4 (0.56%)	0	0
*GJB2*	NM_004004	p.F191L	c.571T>C	0	0	1 (0.19%)	1 (0.37%)
*GJB2*	NM_004004	p.E187_K188delins EKTVFTVFMIAVSGIX	c.558_559ins46	2 (0.14%)	2 (0.28%)	0	0
*GJB2*	NM_004004	p.A171fs	c.511_512insAACG	5 (0.35%)	5 (0.70%)	0	0
*GJB2*	NM_004004	p.R143W	c.427C>T	24 (1.67%)	22 (3.07%)	0	0
*GJB2*	NM_004004	p.G45E,Y136X	c.134G>A,408C>A	35 (2.44%)	32 (4.46%)	1 (0.19%)	1 (0.37%)
*GJB2*	NM_004004	p.F106Y	c.317T>A	1 (0.07%)	1 (0.14%)	0	0
*GJB2*	NM_004004	p.H100fs	c.299_300delAT	10 (0.70%)	9 (1.25%)	0	0
*GJB2*	NM_004004	p.T86R	c.257C>G	11 (0.77%)	10 (1.39%)	0	0
*GJB2*	NM_004004	p.L79fs	c.235delC	122 (8.51%)	90 (12.55%)	3 (0.56%)	3 (1.12%)
*GJB2*	NM_004004	p.I71T	c.212T>C	0	0	1 (0.19%)	1 (0.37%)
*GJB2*	NM_004004	p.G59fs	c.176_191del16	19 (1.32%)	18 (2.51%)	0	0
*GJB2*	NM_004004	p.V37I	c.109G>A	36 (2.51%)	29 (4.04%)	4 (0.74%)	4 (1.49%)
*SLC26A4*	NM_000441	p.P123S	c.367C>T	1 (0.07%)	1 (0.14%)	0	0
*SLC26A4*	NM_000441	p.M147V	c.439A>G	1 (0.07%)	1 (0.14%)	0	0
*SLC26A4*	NM_000441	splice site	c.601-1G>A	5 (0.35%)	5 (0.70%)	0	0
*SLC26A4*	NM_000441	p.V306fs	c.916dupG	1 (0.07%)	1 (0.14%)	0	0
*SLC26A4*	NM_000441	splice site	c.919-2A>G	4 (0.28%)	3 (0.42%)	0	0
*SLC26A4*	NM_000441	splice site	c.1001+1G>A	1 (0.07%)	1 (0.14%)	0	0
*SLC26A4*	NM_000441	p.N392Y	c.1174A>T	1 (0.07%)	1 (0.14%)	0	0
*SLC26A4*	NM_000441	p.T410M	c.1229C>T	15 (1.05%)	12 (1.67%)	1 (0.19%)	1 (0.37%)
*SLC26A4*	NM_000441	p.G439R	c.1315G>A	2 (0.14%)	2 (0.28%)	0	0
*SLC26A4*	NM_000441	p.T527P	c.1579A>C	3 (0.21%)	3 (0.42%)	0	0
*SLC26A4*	NM_000441	p.F550fs	c.1648dupT	1 (0.07%)	1 (0.14%)	0	0
*SLC26A4*	NM_000441	p.V659L	c.1975G>C	1 (0.07%)	1 (0.14%)	0	0
*SLC26A4*	NM_000441	p.T721M	c.2162C>T	3 (0.21%)	2 (0.28%)	0	0
*SLC26A4*	NM_000441	p.H723R	c.2168A>G	37 (2.58%)	31 (4.32%)	2 (0.37%)	2 (0.74%)
*CDH23*	NM_052836	p.P240L	c.719C>T	28 (1.95%)	24 (3.35%)	2 (0.37%)	2 (0.74%)
*CDH23*	NM_001171930	p.E956K	c.2866G>A	2 (0.14%)	2 (0.28%)	0	0
*CDH23*	NM_022124	p.R1417W	c.4249C>T	2 (0.14%)	1 (0.14%)	2 (0.37%)	2 (0.74%)
*CDH23*	NM_022124	p.R1588W	c.4762C>T	13 (0.91%)	13 (1.81%)	0	0
*CDH23*	NM_022124	p.Q1716P	c.5147A>C	3 (0.21%)	3 (0.42%)	0	0
*CDH23*	NM_022124	p.R2029W	c.6085C>T	8 (0.56%)	7 (0.98%)	1 (0.19%)	1 (0.37%)
*CDH23*	NM_022124	p.N2287K	c.6861T>G	1 (0.07%)	1 (0.14%)	0	0
*KCNQ4*	NM_004700	p.Q71fs	c.211delC	8 (0.56%)	8 (1.12%)	0	0
*KCNQ4*	NM_004700	p.F182L	c.546C>G	6 (0.42%)	6 (0.84%)	1 (0.19%)	1 (0.37%)
*TMPRSS3*	NM_032404	p.A260T	c.778G>A	2 (0.14%)	2 (0.28%)	0	0
*TMPRSS3*	NM_032405	p.R106C	c.316C>T	0	0	1 (0.19%)	1 (0.37%)
*TMPRSS3*	NM_032405	p.F71S	c.212T>C	12 (0.84%)	12 (1.67%)	1 (0.19%)	1 (0.37%)
*OTOF*	NM_194323	p.R1172Q	c.3515G>A	9 (0.63%)	8 (1.12%)	0	0
*OTOF*	NM_194248	p.Y474X	c.1422T>A	3 (0.21%)	3 (0.42%)	0	0
*OTOF*	NM_194248	p.R425X	c.1273C>T	1 (0.07%)	1 (0.14%)	0	0
*MYO15A*	NM_016239	p.L3138Q	c.9413T>A	1 (0.07%)	1 (0.14%)	4 (0.74%)	4 (1.49%)
*MYO15A*	NM_016239	p.L3160F	c.9478C>T	9 (0.63%)	9 (1.25%)	0	0
*WFS1*	NM_006005	p.A616S	c.1846G>T	1 (0.07%)	1	0	0
*WFS1*	NM_006005	p.A684V	c.2051C>T	1 (0.07%)	1 (0.14%)	0	0
*WFS1*	NM_006005	p.P724L	c.2171C>T	0	0	1 (0.19%)	1 (0.37%)
*WFS1*	NM_006005	p.D729N	c.2185G>A	1 (0.07%)	1 (0.14%)	0	0
*WFS1*	NM_006005	p.E864K	c.2590G>A	2 (0.14%)	2 (0.28%)	0	0
*COCH*	NM_004086	p.I372T	c.1115T>C	3 (0.21%)	3 (0.42%)	0	0
*ACTG1*	NM_001614	p.G268S	c.802G>A	1 (0.07%)	1 (0.14%)	0	0
*ACTG1*	NM_001614	p.E241K	c.721G>A	1 (0.07%)	1 (0.14%)	0	0
*ACTG1*	NM_001614	p.K118M	c.353A>T	1 (0.07%)	1 (0.14%)	0	0
*TECTA*	NM_005422	p.R491C	c.1471C>T	0	0	1 (0.19%)	1 (0.37%)
*TECTA*	NM_005422	p.T562M	c.1685C>T	0	0	1 (0.19%)	1 (0.37%)
*TECTA*	NM_005422	p.H1400Y	c.4198C>T	1 (0.07%)	1 (0.14%)	1 (0.19%)	1 (0.37%)
*TECTA*	NM_005422	p.T1866M	c.5597C>T	1 (0.07%)	1 (0.14%)	0	0
*CRYM*	NM_001888	p.K314T	c.941A>C	2 (0.14%)	2 (0.28%)	0	0
Mitochondrial 12S rRNA	NC_012920		m.1555A>G	-	11 (1.53%)	0	0
Mitochondrial tRNALeu	NC_012920		m.3243A>G	-	14 (1.95%)	0	0
Mitochondrial tRNASer	NC_012920		m.7445A>G	-	1 (0.14%)	0	0
Mitochondrial tRNALys	NC_012920		m.8296A>G	-	1 (0.14%)	0	0
Total				468 (32.64%)	430	29 (5.39%)	29

### Diagnostic rate and clinical features

We identified 316 SNHL patients who carried at least one mutation in the selected deafness genes (44%), and diagnosed 212 patients with hearing loss of specific genetic cause (30%) among the 717 hearing loss patients. As shown in Table 2, this genetic testing enabled us to identify the genetic cause of hearing loss in 21%, 43% and 30% of patients when segregated into autosomal dominant or mitochondrial, autosomal recessive or sporadic SNHL, respectively, among the diagnosed 212 patients. When classified by severity of hearing loss, the diagnostic rate was 43% among those categorized with profound, which is relatively high, but only15% in those categorized with mild SNHL. When classified by age-of-onset as early childhood (<6 year) or late (>6 year), the diagnostic rate was 41% in younger category, but only 16% in the late-onset cases.

**Table 2:** Diagnostic rate and clinical features.

	Diagnosis (n)	Total (n)	Diagnostic rate
Inheritance			
Autosomal dominat or Mitochondrial	31	151	21%
Autosomal recessive	49	114	43%
Sporadic	81	267	30%
Not provided		185	
Severity of hearing loss			
Mild (21-40dB)	15	100	15%
Moderate (41-70dB)	42	193	22%
Sever (71-90dB)	20	80	25%
Profound (91dB-)	59	136	43%
Not provided		208	
Onset of hearing loss			
Early (0–6 y.o.)	108	261	41%
Late (>6 y.o.)	29	178	16%
Not provided		278	

**Fig 1:**
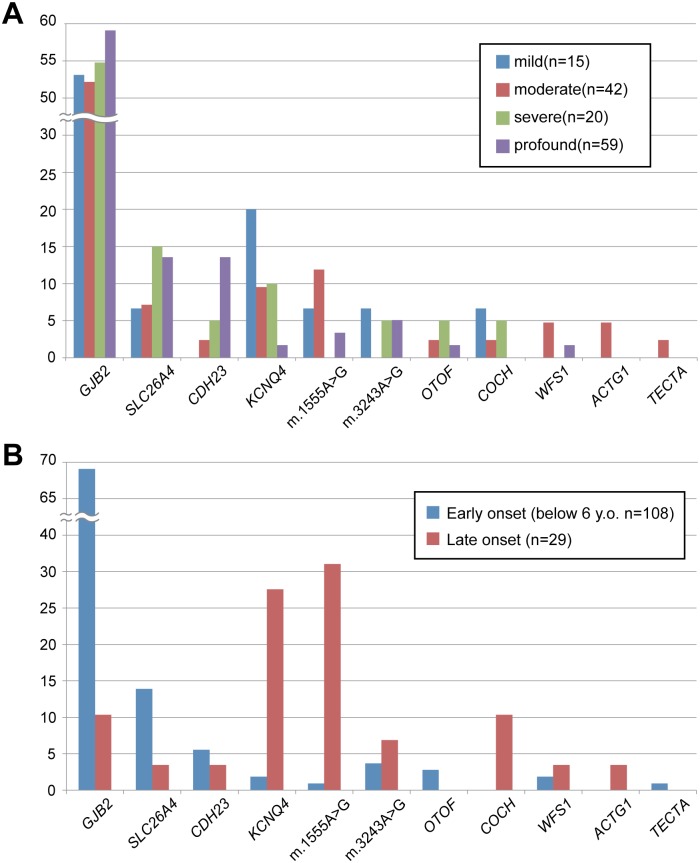
The distribution of genes identified by hearing loss severity (A) and age-of-onset (B). blue: mild, red: moderate, green: severe, purple: profound.

Fig 1 shows the distribution of number of patients with mutations in each gene according to hearing loss severity and age-of-onset. *GJB2* mutations were identified in patients with hearing loss ranging from mild to profound. *SLC26A4* and *CDH23* mutations were frequently identified in patients with severe to profound hearing loss, whereas *KCNQ4* gene mutations and m.3243A>G mutations were frequently identified in patients with hearing loss ranging from mild to moderate. Regarding the onset of hearing loss, mutations in *GJB2* and *SLC26A4* were principally found in the early-onset group. In contrast, most of the patients with mutations in *KCNQ4*, *COCH* or mitochondrial DNA were identified as late-onset.

### Frequency of multiple mutations

As shown in Table 3, 27 patients (3.8% = 27/717) carried multiple mutations in multiple genes. In particular, mitochondrial mutations and mutations in the *GJB2* gene were frequently observed. Of these 27 patients, 8 had mitochondrial mutations together with mutations in other deafness genes, and 6 carried bi-allelic mutations in the *GJB2* gene together with mutations in other deafness genes.

**Table 3:** Cases with multiple mutations.

*Causative mutation(s)*	*Coexistent mutation(s)*	*Number of patients*	*Patient ID*
Mitochondria m.1555A>G; *CDH23*: p.[V1417W];[V1417W]		1	HL0718
Mitochondria m.3243A>G; *KCNQ4*: p.[F182L]	*GJB2*: p.[G45E],[Y136X]	1	HL0239
*CDH23*: p.[P240L];[P240L]; *KCNQ4*: p.[F182L]		1	HL0813
*CDH23*: p.[Q1716P];[R2029W]; *CRYM*: p.[K314T]		2	HL0349, HL400
Mitochondria m.1555A>G	*GJB2*: p.[R143W]	1	HL0257
Mitochondria m.1555A>G	*SLC26A4*: p.[H723R]	1	HL0263
Mitochondria m.3243A>G	*TMPRSS3*: p.[F71S]	2	HL0041, HL0613
Mitochondria m.3243A>G	*SLC26A4*: p.[H723R]	1	HL0086
Mitochondria m.3243A>G	*MYO15A*: p.[L3160F]	1	HL0795
Mitochondria m.3243A>G	*CDH23*: p.[P240L]	1	HL0883
*GJB2*: c.[235delC];[235delC]	*OTOF*: p.[Y474X]	1	HL0222
*GJB2*: c.[235delC];p.[R143W]	*OTOF*: p.[R425X]	1	HL0369
*GJB2*: c.[235delC];[235delC]	*MYO15A*: p.[L3160F]	1	HL0290
*GJB2*: p.[V37I];[R143W]	*TMPRSS3*: p.[F71S]	1	HL0503
*GJB2*: c.[235delC];p.[R143W]	*MYO15A*: p.[L3160F]	1	HL0633
*GJB2*: c.[235delC];p.[R143W]	*TMPRSS3*: p.[F71S]	1	HL0750
*KCNQ4*: p.[F182L]	*GJB2*: p.[F106L]	1	HL0706
*KCNQ4*: p.[F182L]	*MYO15A*: p.[L3160F]	1	HL0415
*SLC26A4*: c.[1001+1G>A];p.[T410M]	*TMPRSS3*: p.[F71S]	1	HL0375
*SLC26A4*: c.[1648dupT];p.[H723R]	*GJB2*: p.[V37I]	1	HL0562
*SLC26A4*: c.[601-1G>A];p.[H723R]	*CDH23*: p.[R1588W]	1	HL0909
	*GJB2*: c.[235delC]; *MYO15A*: p.[L3160F]	1	HL0761
	*GJB2*: c.[235delC]; *TMPRSS3*: p.[F71S]	1	HL0542
	*GJB2*: p.[M195V]; *SLC26A4*: p.[T527P]	1	HL0234
	*GJB2*: p.[V37I]; *CDH23*: p.[P240L]	1	HL0618
Total		27	

Five of the 27 patients (in total 0.7%, 5/717 overall) were diagnosed with genetic hearing loss resulting from concomitant responsible mutations in more than two different genes.

## Discussion

We clarified the allele frequency of mutations identified by the simultaneous screening of 154 mutations in 19 deafness genes in 717 Japanese hearing-loss patients. The overall diagnostic rate of the present screening platform was 30% (212/717). Shearer et al. surveyed 20 reports including 603 individuals with hearing loss of unknown causes tested with MPS and showed that the overall diagnostic rate was 41% (range, 10%-83%), although the rate varied according to various factors, such as sampling selection bias as well as the presence or absence of pre-screening [12]. Using the same platform as that described in this paper, we have recently reported that the diagnostic rate of 52 consecutive deafness subjects was 40% (21/52)[4]. This indicates the diagnostic rate of our current diagnostic platform using non-bias (consecutive) samples is comparable to those of previous reports. The reason for the lower diagnostic rate in this study could be due to the focus on a limited number of selected gene mutations. As the testing is covered by the social health insurance, we selected 154 mutations which have already been reported in literature, and did not include novel identified variants in the diagnostic rate in this study. In clinical settings, uncertain pathogenic variants should be treated carefully. Currently, in a clinical setting, we do not return results for those uncertain variants to the patients directly, but make an additional efforts to evaluate whether those novel variants are pathogenic or not by segregation analysis. For this reason, we first selected 154 mutations in 19 genes covered by social health insurance-based genetic testing. Using this platform, which includes 63 known deafness genes, we can expand the number of mutations if novel variants are proven to pathogenic. With regard to cost, this social health insurance-based genetic testing is much cheaper than 4 comprehensive genetic tests for hearing loss currently available in the United States (350USD vs. Oto Genetics Deafness Test 596USD, Oto SCOPE 1500USD, Oto Seq 3625USD, Oto Genome 3800USD) [12].

With regard to clinical features, when classified by inheritance, severity of hearing loss and age-of-onset, the diagnostic rate was the highest in autosomal recessive, profound hearing loss and early onset cases, respectively. This result reflects the fact that the most frequently identified diagnostic gene was *GJB2*, and these features are consistent with those of *GJB2* mutations.

The frequency of multiple mutations identified by our screening of 154 mutations in 19 genes was 3.8%(27/717), and 5 patients were diagnosed with hereditary hearing loss resulting from multiple responsible mutations (0.7%, 5/717). Usami et al. reported that the frequency of combined (multiple) mutations identified by the Invader assay was 1.5%(4/264)[5]. Haung et al. reported a case who had double bi-allelic mutations in *GJB2* and *SLC26A4* from among 5964 Chinese hearing-impairment patients [13] and 6 cases who had co-existing mutations in *GJB2* or *SLC26A4* and a mitochondrial gene from among 5934 hearing-impairment Chinese patients (0.10%, 6/5934) [14]. The frequency of multiple mutations in this report was higher as we increased the number of screening mutations and genes covered by the screening.

The phenotypes of the cases with multiple causative mutations identified in this study seemed to reflect the phenotype of a more severe form of hearing loss (data not shown). However, when performing genetic counseling, it should be noted that many unknown genes and mutations might affect phenotypes. We previously found that cases with a mitochondrial m.1555A>G mutation in combination with heterozygous *GJB2* mutations show more severe hearing loss than do cases without *GJB2* mutations [15,16]. Moteki et al. reported a hearing loss family caused by a *P2RX2* mutation with a coexisting mit.3243A>G. They suspected that decreases in ATP production due to MELAS with mit.3243A>G might suppress the activation of P2X2 receptors [17]. Further, MPS has revealed the existence of digenic inheritance disease [18,19], and this phenomenon may have some synergic effect on the phenotypes of each patient. Autosomal recessive sensorineural hearing loss should have two mutations in different alleles of one gene and the risk of recurrence was 25% in any sibling. However, it is necessary to note that the risk of recurrence increases in patients with multiple mutations. Therefore, the screening of only one gene, such as *GJB2* direct sequencing, is likely to underestimate the risk of recurrence as well as mislead researchers on novel phenotypes caused by the combination of multiple gene mutations.

Our study suggested that in simultaneous genetic testing of hearing loss was efficient and useful for the detection of pathogenic variants in multiple genes.

## Conclusion

Our study suggested that this social health insurance-based genetic testing protocol was efficient in identifying responsible genes, We clarified the frequency of cases with multiple mutations in different genes and suggested that it was necessary to consider the fact that several genes might have an impact on phenotype in such cases.

## Supporting Information

S1 Table Mutations identified in Japanese deafness patients and reported in the literature. (XLSX)

## References

[1] Smith RJ, Baler JF Jr, White KR. Sensorineural hearing loss in children. Lancet. 2005;365(9462): 879–890. 15752533 10.1016/S0140-6736(05)71047-3

[2] Morton CC, Nance WE. Newborn hearing screening: a silent revolution. The N Eng J Med. 2006;354(20): 2151–2164. 10.1056/NEJMra050700 16707752

[3] Hereditary Hearing Loss homepage. http://hereditaryhearingloss.org/. Accessed February 15, 2015.

[4] Sakuma N, Moteki H, Takahashi M, Nishio S, Arai Y, Yamashita Y, et al An effective screening strategy for deafness in combination with a next-generation sequencing platform: a consecutive analysis. J Hum Genet 2016;61(3):253–261. 10.1038/jhg.2015.143 26763877 PMC4819760

[5] Nishio SY, Hayashi Y, Watanabe M, Usami S. Clinical application of a custom AmpliSeq library and ion torrent PGM sequencing to comprehensive mutation screening for deafness genes. Genet Test Mol Biomarkers. 2015;19(4):209–17. 10.1089/gtmb.2014.0252 25587757 PMC4394162

[6] Usami S, Nishio SY, Nagano M, Abe S, Yamaguchi T, et al Simultaneous screening of multiple mutations by invader assay improves molecular diagnosis of Hereditary hearing loss: a multicenter study. PLoS ONE. 2012;7(2): e31276 22384008 10.1371/journal.pone.0031276 PMC3286470

[7] Miyagawa M, Nishio SY, Ikeda T, Fukushima K, Usami S. Massively parallel DNA sequencing successfully identifies new causative mutations in deafness genes in patients with cochlear implantation and EAS. PLoS One. 2013;8(10): e75793 24130743 10.1371/journal.pone.0075793 PMC3794008

[8] Chang X, Wang K. wANNOVAR: annotating genetic variants for personal genomes via the web. J Med Genet. 2012;49(7): 433–436. 10.1136/jmedgenet-2012-100918 22717648 PMC3556337

[9] Wang K, Li M, Hakonarson H. ANNOVAR: functional annotation of genetic variation from high-throughput sequencing data. Nucleic Acid Res 2010**;** 38(16): e164 10.1093/nar/gkq603 20601685 PMC2938201

[10] Miyagawa M, Nishio SY, Usami S. Prevalence and clinical features of hearing loss patients with *CDH23* mutations. PLoS ONE. 2012;7(8): e40366 10.1371/journal.pone.0040366 22899989 PMC3416829

[11] Nishio SY, Usami S. Deafness gene variations in a 1120 Nonsyndromic hearing loss cohort: molecular epidemiology and deafness mutation spectrum of patients in Japan. Ann Otol Rhinol Laryngol. 2015;124(5S): 49S–60S. 10.1177/0003489415575059 25788563

[12] Shearer AE, Smith RJ. Massively parallel sequencing for genetic diagnosis of hearing loss: the new standard of care. Otolaryngol Head and Neck Surg. 2015;153(2): 175–182. 10.1177/0194599815591156 26084827 PMC4743024

[13] Haung S, Han D, Wan G, Yuan Y, Song Y, Han M, et al Sensorineural hearing loss caused by mutations in two alleles of both *GJB2* and *SLC26A4* genes. Int J Pediatr Otorhinolaryngol. 2013;77(3): 379–383. 10.1016/j.ijporl.2012.11.031 23266159

[14] Haung S, Wang G, Jiang Y, Yuan Y, Han D, Song Y, et al Phenotype and genotype of deaf patients with combined genomic and mitochondrial inheritance models. Mitochondrion. 2013;13(6): 791–794. 10.1016/j.mito.2013.05.004 23688906

[15] Lu SY, Nishio SY, Tsukada K, Oguchi T, Kobayashi K, Abe S, et al Factors that affect hearing level in individuals with the mitochondrial mutation 1555A.G mutation. Clin Genet. 2009;75(5): 480–484. 19475720 10.1111/j.1399-0004.2008.01138.x

[16] Abe S, Kelley PM, Kimberling WJ, Usami S. Connexin 26 gene (*GJB2*) mutation modulates the severity of hearing loss associated with the 1555A>G mitochondrial mutation. Am J Med Genet. 2001;103(4): 334–338. 11746015

[17] Moteki H, Azaiez H, Booth KT, Hattori M, Sato A, Sato Y, et al Hearing loss caused by a P2RX2 mutation identified in a MELAS family with a coexisting mitochondrial 3243AG mutation. Ann Otol Rhinol Laryngol. 2015;124 Suppl 1:177S–183S. 10.1177/0003489415575045 25788561 PMC4441871

[18] Gazzo AM, Daneels D, Cilia E, Bonduelle M, Abramowicz M, Van Dooren S, et al DIDA: A curated and annotated digenic diseases database. Nucleic Acids Res. 2016;44(D1):D900–7. 10.1093/nar/gkv1068. http://dida.ibsquare.be 26481352 PMC4702791

[19] Yoshimura H, Iwasaki S, Nishio SY, Kumakawa K, Tono T, Kobayashi Y, et al Massively parallel DNA sequencing facilitates diagnosis of patients with Usher syndrome type 1. PLoS ONE. 2014;9(3): e90688 10.1371/journal.pone.0090688. 24618850 PMC3949687

